# Green synthesis of silver nanoparticles and characterization of their inhibitory effects on AGEs formation using biophysical techniques

**DOI:** 10.1038/srep20414

**Published:** 2016-02-02

**Authors:** Jalaluddin M. Ashraf, Mohammad Azam Ansari, Haris M. Khan, Mohammad A. Alzohairy, Inho Choi

**Affiliations:** 1 School of Biotechnology, Yeungnam University, Gyeongsan, Republic of Korea; 2 Nanotechnology and Antimicrobial Drug Resistance Research Laboratory, Department of Microbiology, Jawaharlal Nehru Medical College & Hospital, Aligarh Muslim University, Aligarh-202002, U.P., India; 3 Department of Medical Laboratories, College of Applied Medical Science, Buraydah Colleges, Buraydah 51452, Saudi Arabia; 4 Department of Medical Laboratories, College of Medical Science, Qassim University, Saudi Arabia

## Abstract

Advanced glycation end-products (AGEs) resulting from non-enzymatic glycation are one of the major factors implicated in secondary complications of diabetes. Scientists are focusing on discovering new compounds that may be used as potential AGEs inhibitors without affecting the normal structure and function of biomolecules. A number of natural and synthetic compounds have been proposed as AGE inhibitors. In this study, we investigated the inhibitory effects of AgNPs (silver nanoparticles) in AGEs formation. AgNPs (~30.5 nm) synthesized from Aloe Vera leaf extract were characterized using UV-Vis spectroscopy, energy-dispersive X-ray spectroscopy (EDX), high resolution-transmission electron microscopy, X-ray diffraction and dynamic light scattering (DLS) techniques. The inhibitory effects of AgNPs on AGEs formation were evaluated by investigating the degree of reactivity of free amino groups (lysine and arginine residues), protein-bound carbonyl and carboxymethyl lysine (CML) content, and the effects on protein structure using various physicochemical techniques. The results showed that AgNPs significantly inhibit AGEs formation in a concentration dependent manner and that AgNPs have a positive effect on protein structure. These findings strongly suggest that AgNPs may play a therapeutic role in diabetes-related complications.

The Maillard reaction is a nonenzymatic reaction of reducing sugars with amino groups of biological macromolecules. This process, which is also known as glycation, involves post-translation protein modification and may be responsible for a variety of diseases. The reaction is initiated by the reversible formation of a Schiff base between a reducing sugar and the amino group of a protein, DNA and lipoproteins^1^,^2^,^3^. The relatively unstable Schiff base undergoes rearrangement to form a more stable Amadori product, which in turn undergoes a series of reactions to form advanced glycation end products (AGEs)^4^,^5^. The accumulation of these AGEs in long-lived tissue is thought to be involved in diabetic complications and aging^6^.

The Maillard reaction is found to be instigated by several sugar and non-sugar metabolites. Methylglyoxal (MG) is one of the most reactive metabolites that are involved in the formation of AGEs. It is generated during several enzymatic and nonenzymatic processes like glycolytic pathway, autoxidation of sugars and during all stages of the Maillard reaction^7^,^8^. Very high MG concentration has been detected in the lens, blood and kidney of diabetic patients^9^. For instance, 5–6 and 2–3 fold increases of MG was noted in Type I and II diabetic patients, respectively, as compared to their normal counterparts^7^,^9^. Considering its high reactivity with proteins and presence of significant amounts of MG in the plasma (0.1 mM), MG may play as one of the major glycating agents in the body^10^. Moreover, it was found that MG glycated the receptor proteins located on the surface of cytoplasmic membrane of macrophages^11^.

Since AGEs contribute to the onset of several diseases, including diabetic complications^12^, inhibitors to prevent the formation of AGEs have been extensively investigated over the last few years to minimize their involvement in diseases. Notable potential anti-glycating agents have been reported, including aminoguanidine^13^, aspirin^14^, vitamin B6^15^, taurine^16^, quercetin^17^ and anti-inflammatory drugs such as ibuprofen^18^. Nanotechnology, an interdisciplinary research field involving chemistry, engineering, biology, and medicine, has great potential for early detection, accurate diagnosis and personalized treatment of cancer and other diseases^19^.

Nanoparticles (NPs), which are 100 to 10,000 times smaller than human cells, offer unprecedented interactions with biomolecules on both the surface and inside of the cells. AgNPs have been used for numerous physical, biological, and pharmaceutical applications because their small size and similarity to cellular components enables them to enter living cells using cellular endocytosis mechanisms, especially pinocytosis^20^. Interestingly, AgNPs have been reported to exhibit antibiofilm^21^, anticancer^22^, antibacterial^23^,^24^ antimicrobial^25^, anti-inflammatory and anti-oxidant activities^26^,^27^,^28^. A previous study showed that silver nanoparticles (AgNPs) were potential inhibitors of AGEs formation^29^.

This study was conducted to provide direct evidence of the inhibitory strength of AgNPs in HSA (human serum albumin) glycation using various physicochemical techniques. This information was obtained by the detection of AGE-absorbance and fluorescence, estimation of CML, side chain modification of HSA and study of the secondary structure of HSA after incubation with MG in the presence or absence of varying concentrations of AgNPs.

## Materials and Methods

### Preparation of the leaf extract

Aloe vera was selected for the biosynthesis of AgNPs because of its cost effectiveness, ease of availability and medicinal properties. Biosynthesis was conducted as previously described, with minor modifications^30^. Fresh and healthy leaves were collected locally and rinsed thoroughly with tap water followed by doubled distilled water to remove all dust and unwanted visible particles, after which they were dried at room temperature to remove the water from the surface of leaves, then cut into small pieces. Next, 10 g of these finely incised leaves were transferred into 250 ml beakers containing 100 ml distilled water and boiled at 80 °C for 20 min. After cooling at room temperature, leaves were centrifuged at 12,000 rpm for 15 min at 4 °C and filtered through 0.45 μm PTFE filter. The filtrates were then stored at 4–8 °C and used as reducing and stabilizing agents in the synthesis of AgNPs. Sterility was maintained throughout the experiment^30^.

### AgNPs synthesis

Aqueous solution of 1 mM silver nitrate (AgNO_3_) was prepared in a 250 ml Erlenmeyer flask and used for the synthesis of AgNPs. Briefly, 10 ml of *Aloe vera leaf extract* was added into 90 ml of aqueous solution of 1 mM silver nitrate and incubated in the dark overnight at room temperature. Complete reduction of AgNO_3_ to Ag^+^ ions was confirmed by the change in colour from colorless to colloidal brownish yellow. The colloidal mixture was then sealed and stored properly for future use. The formation of AgNPs was further confirmed by spectrophotometric analysis.

### UV-Vis spectra analysis

Preliminary characterization of the AgNPs was carried out using UV–Visible spectroscopy. The reduction of silver ions to the nanoparticle form was monitored by measuring the UV–Visible spectra of solutions after diluting the sample with Millipore water 20 times. The spectra of AgNPs solution was monitored by a UV-Vis spectrophotometer (Varian Inc., USA) from 300 to 600 nm. Millipore water was used as blank to adjust the baseline.

### X-ray diffraction (XRD) analysis

XRD analysis of AgNPs was performed as described by Ansari *et al.*^31^. The XRD pattern of AgNPs was recorded by a Bruker D8 diffractometer using CuK_α_ radiation (λ = 1.54056 Å) in the range of 20° ≤ 2θ ≤ 80° at 40 keV. The lattice parameters were calculated by the PowderX software. The particle size (*D*) of the sample was calculated using the Scherrer’s relationship:


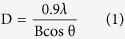


where, *λ* is the wavelength of the x-ray, *B* is the broadening of the diffraction line measured as half of its maximum intensity in radians and *θ* is the Bragg’s diffraction angle. The particle size of the sample was estimated from the line width of the (111) XRD peak.

### High-resolution transmission electron microscopy (HR-TEM) and dynamic light scattering (DLS) analysis of AgNPs

The size and morphology of the synthesized nanoparticles were further analyzed by HR-TEM. Samples were prepared by placing a drop of a very dilute suspension of nanoparticles solution on a carbon-coated copper grid. The samples were allowed to dry by evaporation at ambient temperature and then kept under a desiccator until loading onto a specimen holder. The TEM measurements were performed at an accelerating voltage of 200 kV (Technai G2, FEI, Electron Optics, USA) with a W-source and an ultra high resolution pole piece. Dynamic light scattering was used to determine the size distribution or average sizes of synthesized silver nanoparticles.

### Chemicals

Crystallized and fatty acid free human serum albumin (HSA), MG, and dialysis tubing were purchased from Sigma Chemical Company, USA. Sodium monobasic and dibasic salts were purchased from Quali-gens, India. Unless otherwise indicated, all other reagents and solvents were of analytical grade.

### Experimental mixtures preparation

The glycation procedure involves the use of HSA, MG as a glycating agent and AgNPs as an AGEs inhibitor in the reaction system. Briefly, HSA (100 μM) and MG (1 mM) were mixed with or without varying concentrations (0.09, 0.18 and 0.27 mM) of AgNPs in 0.05 M phosphate buffered saline (PBS, pH 7.4), after which mixtures were incubated for 6 days at 37 °C. Native HSA (without MG and AgNPs) was used as a control. After day 6 of incubation, the solutions were dialyzed against sodium phosphate buffer at 4 °C for 48 h to remove the unbound MG and AgNPs from solution. Following dialysis, samples were appropriately diluted and used immediately for analysis.

### Determination of free amino groups by fluorescamine

Lysine side chain modifications were monitored using fluorescamine as described previously, with slight modification^32^. This process forms a highly fluorescent reaction product with amino groups. G-modified HSA with or without AgNPs solution (5 μl; approx. 1 mg/ml), 100 μl of 100 mM Na_2_HPO4, 45 μl aqua dest, and 50 μl fluorescamine reagent (1 mM fluorescamine in acetonitrile) were mixed and incubated for 10 min in the dark in a 96-well plate. The fluorescence of the sample was then measured at excitation/emission wavelengths of 390/490 nm in a FLUORO-STAR plate reader (BMG, Germany). N-α-acetyl-lysine from 0 to 1.5 mM was used to determine the linearity of fluorescence within the expected lysine content of the protein solution.

### Determination of free arginine side chains by 9,10-phenanthrenequinone

Free arginine was determined as previously described^33^. Briefly, 50 μl samples were mixed in 150 μl of 9,10-phenanthrenequinone reagent (150 μM in ethanol) and 25 μl of 2 N NaOH. Protein samples without 9,10-phenanthrenequinone were used to correct the results for AGE fluorescence. Samples were incubated at 60 °C for 3 h, after which 40 μl were transferred to a 384 well plate and mixed with 40 μl of 1.2 N HCl. Fluorescence in the reaction product was allowed to develop for 1 h in the dark at room temperature, then measured with a TECAN Safire spectrometer (USA) at excitation/emission wavelengths of 312/395 nm. To test the linearity of fluorescence within the expected arginine content of the protein solution, N-α-acetyl- arginine from 0 to 0.4 mM was used.

### Estimation of protein-bound carbonyl contents

Carbonyl contents of native and MG-modified HSA with or without AgNPs samples were determined as previously described, with slight modifications^34^. Briefly, 15 μM native and MG-modified HSA with or without AgNPs samples dissolved in 10 mM DNPH (2,4-dinitrophenyl hydrazine) solution was made in 2 N HCl. Samples were then vortexed for 1 h at room temperature and precipitated with 0.5 ml of 20% (v/v) TCA, followed by 3 min of centrifugation at 11,000 g at 4 °C. The pellet was washed with 1 ml of ethanol-ethyl acetic acid mixture (1:1; v/v) to remove extra DNPH reagent. Next, samples were incubated at room temperature for 10 min and then centrifuged at 11,000 × g for 5 min at 4 °C. The supernatant was discarded and the pellet was washed twice with ethanol-ethyl acetic acid mixture. The protein pellet was then suspended in 1 ml of 6 M guanidium hydrochloride dissolved in 20 mM potassium phosphate buffer, pH 2.3 (attuned with trifluoroacetic acid), after which samples were incubated at 37 °C for 15–30 min to ensure complete solubility of proteins. All samples were subsequently centrifuged to remove any insoluble material. Carbonyl content was determined in the supernatant based on the absorbance at 370 nm against 6 M guanidium hydrochlorides (as blank) using the molar extinction coefficient of 22,000 M^−1^ cm^−1^. Protein carbonyl content was expressed as nmol/mg of protein.

### Estimation of carboxymethyl lysine (CML) content by ELISA

CML content was measured by ELISA as described earlier, with minor modification^35^. Briefly, absorbance was measured at 405 nm using a Model 550 BioRad microplate reader. Product formation was then measured with a 405 nm filter in an ELX800 multiwell plate reader (BioTek Instruments, USA).

### UV-Visible and fluorescence spectroscopy

UV-visible absorption spectra of native and treated HSA were carried out using a Cary Win UV-Vis spectrophotometer (Varian Inc., USA) in the range of 250–500 nm. Fluorescence spectra were obtained on a Jasco FP-6500 (Japan). Samples were positioned in a cuvette with a 1 cm path length. All emission spectra were recorded with 1 nm wavelength intervals. Native HSA and glycated-HSA emission spectra were obtained in the range of 360–600 nm under excitation at 360 nm.

To calculate the percent inhibition of AGEs formation by various concentrations of AgNPs, the MG-modified sample was used as a positive control. The percent inhibition was calculated using the following formula:


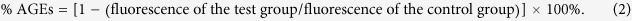


### High performance liquid chromatography (HPLC)

HPLC analysis was performed on a Hitachi analytical HPLC system (Japan) composed of L-7100 low-pressure gradient pumps, an L-7200 sequential auto-sampler and a high sensitivity diode array detector (190–800 nm) that was managed by a D-7000 HPLC System Manager software. The products formed in the incubated mixtures of HSA and MG were identified on a Phenomenex Luna 5 u, 100 Å, HPLC column (150 mm × 4.6 mm × 5 μm). Mobile phases A and B were 0.1 M aqueous ammonium acetate and 100% HPLC analytical grade acetonitrile, respectively. Aqueous ammonium acetate solution was prepared in deionized water and both mobile phases were degassed and sonicated for 15 min before use. Prior to HPLC analysis, all samples and solvents were filtered with Millipore 0.22 μm syringe filters. The accuracy and reproducibility of the HPLC method was confirmed by repeated testing.

### Circular Dichroism (CD)

Far-UV CD profiles of samples were recorded on a Jasco spectropolarimeter (J-815, Japan) attached to a Jasco Peltier-type temperature controller (PTC-424S/15). The instrument was calibrated with D-10-camphorsulphonic acid and measurements were carried out at 25 °C using a temperature controlled cell holder attached to a Neslab’s RTE water bath with a temperature accuracy of ±0.1 °C. Solutions of control and glycated samples with or without AgNPs (20 μM) were placed in a 1 mm pathlength cuvette and profiles were recorded in the wavelength range of 200–250 nm. A scan speed of 100 nm/min and response time of 1 second was chosen to record the CD spectra. Two sets of each sample were studied under identical conditions to confirm reproducibility of the results^36^.

### Fourier transformation infrared (FTIR) spectroscopy

FTIR spectroscopy was used to investigate the changes in structure in MG-modified protein with various concentrations of AgNPs. Buffer subtracted transmission spectra were recorded in the wave number range of 600–4000 cm^−1^ using a Perkin Elmer spectrum 100 FTIR spectrometer (PerkinElmer, Inc., USA).

## Results and Discussion

### Characterization of AgNPs

**Figure 1:**
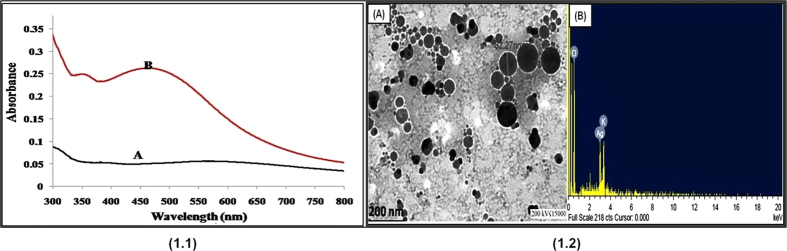
**(1.1)**UV–vis absorption spectrum of (**A**) silver nitrate (1 mM) and silver nanoparticles (**B**) synthesized using extract of fresh Aloe vera leaf.**(1.2)** HR-TEM micrograph (**A**) and EDX spectrum (**B**) of green synthesized silver nanoparticles.

**Figure 2:**
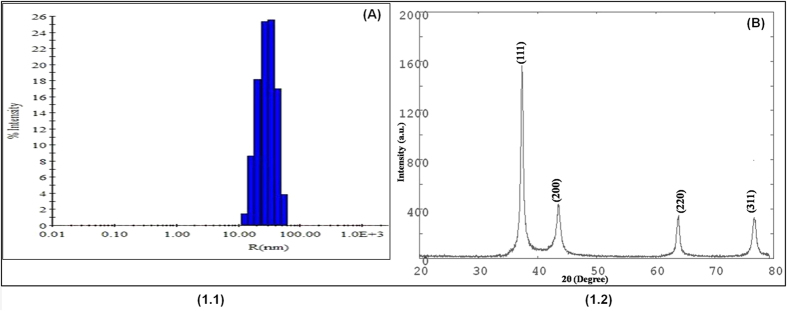
**(1.1)**DLS histogram showing particle size distribution of synthesized silver nanoparticles.**(1.2)** X-ray powder diffraction pattern of green synthesized silver nanoparticles.

Prior to investigation of the antiglycating effect of AgNPs, characterization of synthesized AgNPs was performed as previously described, with slight modifications^31^. Briefly, Aloe Vera leaf extract was used in this study for one point green synthesis of AgNPs at room temperature. Upon adding the leaf extract to the silver nitrate solution, a faint yellow color was observed after 24 h of reaction (avoid light). The appearance of yellow color, which is due to the excitation of surface plasmon vibrations, represents spectroscopic signature of AgNPs formation^37^. Characterization of biosynthesized AgNPs was accomplished using UV-Vis spectroscopy, XRD, DLS and HR-TEM (Figs 1 and 2). In the UV–Visible spectrum a strong broad peak was observed at 455 nm, and widening of the peak indicated that the particles were polydispersed (Fig. 1.1). AgNPs are known to exhibit a UV–Visible absorption maximum in the range of 400–500 nm because of surface plasmon resonance^38^. HR-TEM analysis revealed that the AgNPs were primarily spherical in shape. Their size ranges from in between 5–85 nm (Fig. 1.2), and the size distribution histogram of DLS indicated that the average size was 30.5 nm (Fig.1). The XRD pattern indicated that the nanoparticles had a face-centered cubic structure (JC-PDS No: 03-0921). All peaks of the XRD pattern could be indexed according to the Ag (Fig. 2).

It is well known that blood proteins including albumin are glycated in diabetic patients by a variety of reducing sugars, leading to the formation of AGEs and affecting the structure and function of proteins^39^. Following the addition of reducing sugars, various metabolites react with amino groups of biomolecules. Methylglyoxal (2-oxopropanal) is a reactive physiological metabolite^40^ that is mainly responsible for forming inter- and intra-molecular cross-links of proteins known as AGEs^41^. On the basis of high pervasiveness of AGEs and oxidative stress in numbers of diseases; it has become quite imperative to explore the new inhibitors which can be utilized for the prevention of AGEs formation. The formation of AGEs may be prevented by inhibiting glycoxidation process or by sequestering the reactive α-dicarbonyl compounds. Aminoguanidine and other inhibitors (Pimagedine1) were initially found to be potential inhibitors of Maillard reaction^42^. However, later their use was abandoned due to the side effect observed during phase III clinical trials in diabetic patients^43^. Discovery of new molecules and manipulation of those available that are naturally nanosized has great potential to improve health care^44^. Nanosize particles showing anti-glycating properties may also have important clinical significance. Keeping the above explanation in mind, we attempted to discern the role of AgNPs in the inhibition of AGEs formation.

Epsilon-amino groups in native and MG-glycated HSA were determined by fluorescamine assay. The percentage of reacted lysine residues in the MG-glycated HSA mixture without AgNPs was found to be 66.70% relative to the control. A decrease in the reactivity of lysine residues (50.25, 35.30 and 28.85%) was observed with increasing concentrations (0.09, 0.18 and 0.27 mM) of AgNPs. A similar trend was also observed in the reactivity of arginine residues with increasing concentrations 0.09, 0.18 and 0.27 mM of AgNPs. The MG-glycated HSA mixture with or without Aloe vera leaf extract, the arginine and lysine residues showed almost same reactivity (Table 1). These results show that the reactivity of both lysine and arginine residues of HSA with MG gradually decreases with increasing concentration of AgNPs, indicating that AgNPs are capable of inhibiting the glycation reaction at the initial stage in a concentration dependant-manner.

MG-mediated oxidation may lead to modification of amino acid side chains. Carboxylation of lysine, arginine, threonine and proline residues is a typical marker of protein oxidation. The carbonyl content in MG-glycated HSA mixture with or without Aloe vera leaf extract was (25.83 ± 1.23 and 23.61 ± 1.23 nmol/mg protein respectively) approximately same, but 19.33 ± 1.10, 14.58 ± 1.02 and 8.36 ± 0.08 nmol/mg protein carbonyl contents was observed in the reaction mixture with increasing concentration (0.09, 0.18 and 0.27 mM) of AgNPs, respectively (Table 1). The decreasing carbonyl content with increasing levels of AgNPs was caused by the antiglycating activity of AgNPs. The presence of carbonyl contents *in vivo* and *in vitro* are considered biomarkers of oxidative stress that predict irreversible oxidative modifications in proteins during the glycation process^45^. The CML content in native and MG-glycated HSA protein was determined by ELISA. In native HSA, no CML was observed, while a significant and almost same amount of CML was detected in MG-glycated HSA mixture with or without Aloe vera leaf extract (1.86 ± 0.30 mol/mol HSA and 1.79 ± 0.44 respectively). However, the MG-HSA mixture showed 1.46 ± 0.15, 0.96 ± 0.13 and 0.58 ± 0.08 nmol/ml HSA CML content upon treatment with increasing concentrations of 0.09, 0.18 and 0.27 mM AgNPs, respectively (Table 1). Several types of AGEs and their structures have been described *in vivo* and *in vitro*, among which CML and pentosidine are considered important AGEs^46^,^47^. The oxidatively formed AGEs CML and pentosidine are closely related to oxidative stress-induced damage^48^. Our study showed that formation of CML and carbonyl content decreased with increasing levels of AgNPs, suggesting that AgNPs has substantial ability to inhibit AGEs formation.

**Figure 3:**
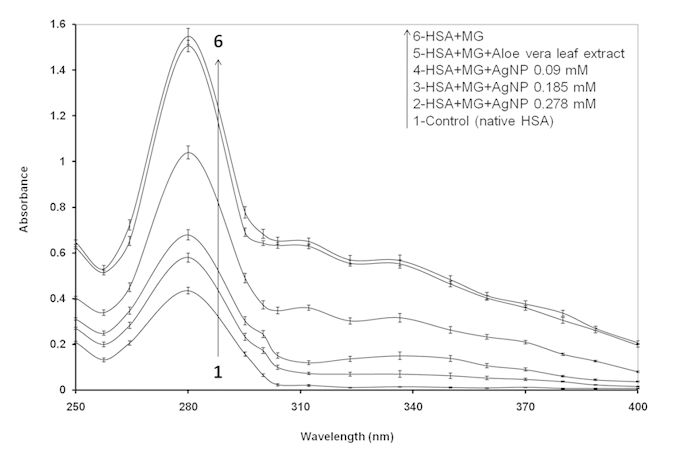
UV absorbance spectra of HSA and MG with varied concentrations of AgNP incubated at 37 °C for 6 days. UV absorbance was monitored at 280 nm.

UV-Visible spectra of native HSA showed the maximum absorbance at 280 nm (Fig. 3). Upon modification with MG, an increase in absorbance (hyperchromicity) was observed at 280 nm. When compared to native HSA, the MG-glycated HSA mixture with or without Aloe vera leaf extract showed approximately 71.90% hyperchromicity at 280 nm, as well as an increase in the absorbance at 300–400 nm. However, when the MG-HSA mixture was incubated with 0.09, 0.18 and 0.27 mM of AgNPs, the hyperchromicity was 58.0%, 35.93% and 25.0%, respectively. Incubation of the MG-HSA mixture with increasing concentrations of AgNPs also led to a significant decrease in absorbance between 300 and 400 nm. Aloe leaf extract did not show significant difference in absorbance at 200–400 nm (Figure S1). The hyperchromicity observed at 280 nm in modified-HSA may be due to exposure of aromatic amino acids or formation of new chromophoric groups resulting from unfolding of the protein helix upon glycation. A previous study attributed the hyperchromicity of protein as a result of glycation^49^, whereas the increase in absorbance from 300 to 400 nm suggests the generation of AGEs^45^,^49^. A number of studies have also explained the usefulness of increases in absorbance in this range (300–400 nm) in determining the formation of proteins-AGEs or DNA-AGEs^50^.

**Figure 4:**
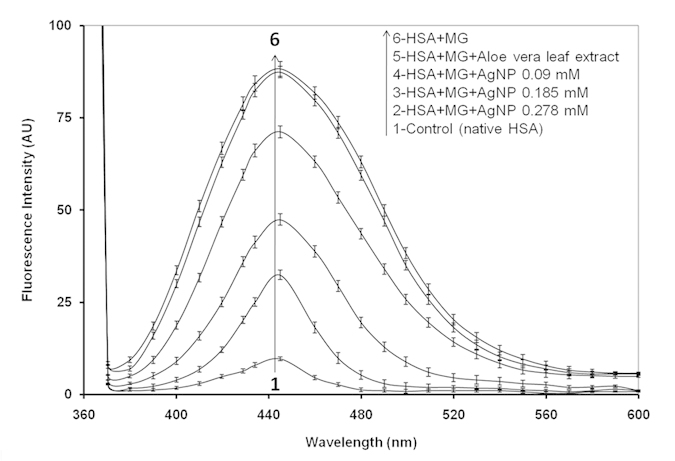
Fluorescence emission intensity profiles of HSA and MG with varied concentrations of AgNP. Fluorescence intensities were measured at excitation and emission wavelengths of 365 nm and 444 nm, respectively.

Native HSA and MG mixtures with various concentrations of AgNPs were excited at λ_ex_ 365 nm, and the emission (λ_em_ = 445 nm) profiles were recorded to determine the inhibitory effects of AgNPs in advanced glycation end products formation. MG-glycated HSA mixture with or with without Aloe leaf extract showed almost same and comprehensively elevated emission fluorescence intensity (84%) at λ_max_ 445 nm. When HSA and MG mixtures were incubated with 0.09, 0.18 and 0.27 mM AgNPs, the fluorescence intensity was observed to be 44.6, 29.3 and 13.7%, respectively, compared to native HSA (Fig. 4). In addition, AgNPs at 0.09 and 0.18 mM inhibited the formation of AGEs induced by MG by 19.36% and 46.31%, respectively, while the highest inhibition (64.0%) of AGEs formation was observed when 0.27 mM AgNPs was used. No inhibition of AGEs formation was when MG-HSA mixture was incubated with Aloe vera leaf extract. Fluorescence intensities of controls and Aloe leaf extract were found to be negligible (Fig. 4; Figure S2). Our results are consistent with those of previous studies as several AGEs exhibit a characteristic fluorescence around at 370 nm/440 nm^51^, and fluorescence intensity can be measured *in vitro* to examine the formation of AGEs under different treatments^52^. The decrease in fluorescence and AGEs formation with increasing concentrations of AgNPs demonstrates that AgNPs act as potential antiglycating agents in a concentration-dependant manner.

**Figure 5:**
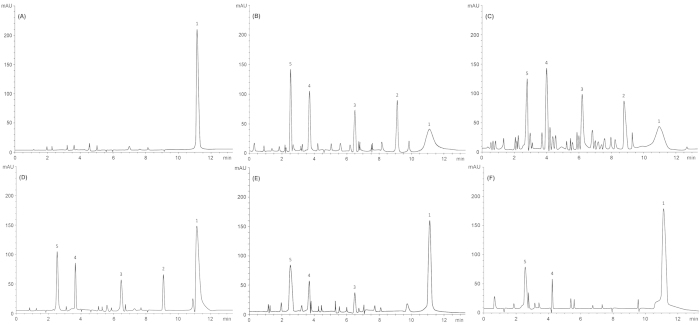
HPLC/UV elution profiles of HSA with MG mixtures with or without AgNP after 6 days of incubation. **(A)** Native HSA, **(B)** HSA–MG mixtures without AgNP, **(C)** HSA–MG with AgNP (0.09 mM), **(D)** HSA–MG with AgNP (0.185 mM), and **(E)** HSA–MG with AgNP (0.27 mM). All readings were taken in triplicate. UV Absorbance was measured at 280 nm.

HPLC elution profiles of native HSA, HSA–MG without AgNP, and HSA–MG with 0.09, 0.18 and 0.27 mM AgNPs after 6 days of incubation at 37 °C are illustrated in Fig. 5A,B,C,D,E and F. Native HSA showed a high intensity peak at a retention time of 11.14 min (Fig. 5A). On incubating the HSA and MG mixture with or without Aloe leaf extract, presence of prominent peaks with retention times of 9.12 (peak 2), 6.5 (peak 3), 3.7 (peak 4) and 2.5 (peak 5) min reveals formation of AGEs species (Fig. 5B,C). The diminution and disappearance of peaks was observed in HSA and MG mixtures incubated with 0.18 and 0.27 mM AgNPs, respectively (Fig. 5D,E). When the mixture of HSA and MG was incubated with 0.09 mM AgNP, only diminution of peaks was observed, whereas no such significant peaks were detected for Aloe vera alone (Fig. 5F; Figure S3). Analysis of the HPLC data revealed that AGEs generation was reduced with systemic augmentation of AgNPs concentration. Overall, assessment of the entire dataset clearly confirmed that AgNPs are a very strong antiglycating agent. The HPLC data substantiated the UV/VIS and fluorescence findings that the formation of AGEs was influenced by AgNPs in a concentration-dependent manner. Previous studies have reported anti-glycation activity of gold nanoparticles (GNP) on hemoglobin and crystalline protein^53^,^54^. In addition, AgNPs were found to decrease the cell permeability of AGEs-modified proteins by stimulating the expression of tight junction proteins^55^. Thus, the results of our study are consistent with previous findings of inhibition of AGEs by nanoparticles.

Far-UV CD spectroscopy can be used to investigate the secondary structure of proteins and their conformational changes. The far-UV CD spectrum of HSA is characterized by the presence of two strong negative bands at 208 and 222 nm, which represent the helical characteristics of HSA^56^. Therefore, the change in ellipticity [*θ*] at 208 and 222 nm was used to monitor the change in helical content of protein samples and secondary structure of HSA.

**Figure 6:**
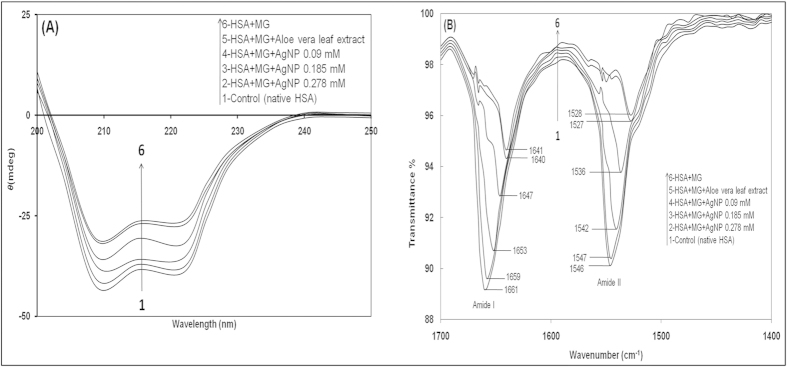
(**A**)CD profiles of HSA–MG with and without various concentrations of AgNPs incubated at 37 °C for 6 days. All samples were appropriately diluted with deionized water before obtaining the CD profile. Repeat studies revealed no significant difference in CD profiles. **(B)** FTIR profiles of Amide I and Amide II bands of HSA and MG mixtures with and without AgNPs after 6 days of incubation. All readings were taken in triplicate.

The CD curve of HSA exhibited a larger divergence at 208 and 222 nm, indicating that HSA had the maximum α-helical content. After incubation for 6 days, the HSA and MG mixture showed marked diminution of divergence at 208 and 222 nm, indicating that reduction of the α-helical content was due to modification of the HSA secondary structure by MG. When compared to CD measurements of the MG-HSA mixture, more α-helical content of the protein was retained in mixtures with increasing levels of AgNPs, indicating that the extent of HSA modification in the presence of AgNPs (0.09, 0.18 and 0.27 mM) was reduced (Fig. 6A). These profiles showed more α-helix content remained in the HSA secondary structure when incubated with 0.18 and 0.27 mM of AgNPs than in mixtures with 0.09 mM of AgNP, indicating that the diminution of structural modification of HSA is a concentration dependent phenomenon. The CD results clearly indicate that AgNPs have a positive effect on α-helixes and help retain the secondary structure of HSA.

The Far-UV CD results were further supported by FT-IR spectroscopy. FTIR spectra of MG-HSA mixtures incubated for 6 days with AgNP were analyzed in the range of 1400–1700 cm^−1^ (Fig. 5B). Secondary structure analysis was based on Amide-I and Amide-II bands within the 1400–2000 cm^−1^ region after subtraction of the background absorption. In the IR region, the amide I peak position occurs in the 1600–1700 cm^−1^ region (mainly C=O stretch), while the amide II band was located from 1500–1600 cm^−1^ (C–N stretch coupled with N–H bending mode), representing the amount of carbonyl and amino bonds in side chains of amino acid residues of the HSA structure^57^. Native HSA showed spectra at 1660.80 and 1547.0 cm^−1^ in the Amide I and II region, respectively. The change in band position and increase in transmission in MG-HSA was due to the HSA reaction with MG (Fig. 6B), which indicates that the altered secondary structural elements evolved due to the glycation reaction. As in the presence of 0.18 and 0.27 mM AgNPs in MG-HSA solution, transmission and band positions (amide I & II) showed minimal changes relative to the 0.09 mM AgNP and MG-HSA reaction mixtures, indicating more unaffected amide bonds and amino groups were present in the solution containing AgNPs^58^. The change in peak positions in the amide I & II regions and transmittance in FTIR spectroscopy demonstrated a change in the secondary structure of HSA after modification with MG. However, the effects of MG on HSA secondary structure were found to gradually decrease with increasing concentrations of AgNPs. Thus, CD and FTIR analysis of the MG-HSA mixture with and without AgNPs corroborate that AgNPs play an important role in maintaining the secondary structure of HSA protein.

Amino acids containing free amino groups (Lys, Arg) are potent sites for glycation in addition to the N-terminal amino acid. AgNPs competitively bind to these free amino groups. These observations gives an indication of decrease in the glycation upon masking of the free amino groups or sequestration of reacting group of glycating agents by AgNPs (e.g., those residing on the lysine residues)^59^,^60^. However, the exact mechanism of action of AgNPs is still not clear. In summary, the results of our study indicate that (i) AgNPs can reduce the rate of non-enzymatic modification of HSA by MG and (ii) AgNPs can change the secondary structure of HSA in a concentration independent manner.

## Conclusion

The search for anti-glycating agents is an important clinical issue, as reactive carbonyl entities such as MG, glyoxal and 3-DG are increasingly impacting biological components such as proteins and DNA of the cellular system. In the past, medicinal plants were primarily explored for their anti-oxidant potential. Now, these organisms are being investigated for the presence of novel inhibitors that can nullify the effect of AGEs and help remove the threat posed by these reactive carbonyls that eventually leads to glycation. Several studies have revealed a broad range of potential antibacterial, antimicrobial, anticancer and anti-oxidative activity of AgNPs. Additionally, AgNPs have been shown to interact with the HIV-1 virus and inhibit its ability to bind host cells^3^. Therefore, AgNPs are believed to have the potential for application for the treatment of several diseases.

The results of our present study illustrate that biosynthesized AgNPs have potential anti-glycating ability that may enable their use as therapeutics in the treatment of diabetes related complications. However, a systematic and comprehensive study of the mechanism and the downstream pathways is required before we can expect a more meaningful role of nanoparticles in medicinal applications.

## Additional Information

**How to cite this article**: Ashraf, J. M. *et al.* Green synthesis of silver nanoparticles and characterization of their inhibitory effects on AGEs formation using biophysical techniques. *Sci. Rep.* **6**, 20414; doi: 10.1038/srep20414 (2016).

## Supplementary Material

Supplementary Information

## Figures and Tables

**Table 1:** Effect of AgNPs on various parameters and AGEs.

Sample	Lysine reacted (%)	Arginine reacted (%)	Carbonyl nmol/mg protein	CML nM/ml HSA
Native HAS (Control)	—	—	3.75 ± 0.28	0
HSA+MG	66.70	59.90	25.83 ± 1.16	1.85 ± 0.30
HSA+MG+Aloe vera Leaf extract	63.73	53.46	23.61 ± 1.23	1.79 ± 0.44
HSA+MG+AgNP 0.09 mM	50.25	40.60	19.33 ± 1.10	1.46 ± 0.15
HSA+MG+AgNP 0.18 mM	35.30	29.75	14.58 ± 1.02	0.96 ± 0.13
HSA+MG+AgNP 0.27 mM	28.85	21.26	8.36 ± 0.08	0.58 ± 0.08
