# Supplementary material for: Green synthesis of silver nanoparticles and characterization of their inhibitory effects on AGEs formation using biophysical techniques
Source: Sci Rep. 2016 Feb 2;6:20414. doi: 10.1038/srep20414 (PMC4735866; doi:10.1038/srep20414)
Supplement: Supplementary Information [file srep20414-s1.pdf]

Supplementary Information for

Green synthesis of silver nanoparticles and characterization of their inhibitory effects on AGEs formation using biophysical techniques

#Jalaluddin M. Ashraf*[1], #Mohammad Azam Ansari*[2, 3], Haris M. Khan[2], Mohammad A. Alzohairy[3], Inho Choi*[1]

# contributed equally to this work

[1]School of Biotechnology, Yeungnam University, Gyeongsan, Republic of Korea

[2]Nanotechnology and Antimicrobial Drug Resistance Research Laboratory, Department of Microbiology, Jawaharlal Nehru Medical College & Hospital, Aligarh Muslim University, Aligarh-202002, U.P., India

[3]Department of Medical Laboratories, College of Applied Medical Science, Buraydah Colleges, Buraydah 51452, Saudi Arabia

Corresponding authors: *Jalaluddin M. Ashraf (jmashraf@gmail.com) or Mohammad Azam Ansari, azammicro@gmail.com or Inho Choi, (inhochoi@ynu.ac.kr)

Supporting Information

Figure S1. UV–vis absorption spectrum of Alove vera leaf extract.

Figure S2. Fluorescence emission intensity profile of Aloe vera leaf extract. Fluorescence intensities were measured at excitation and emission wavelengths of 365 nm and 444 nm, respectively.

Figure S3. HPLC/UV elution profile of Aloe vera leaf extract.

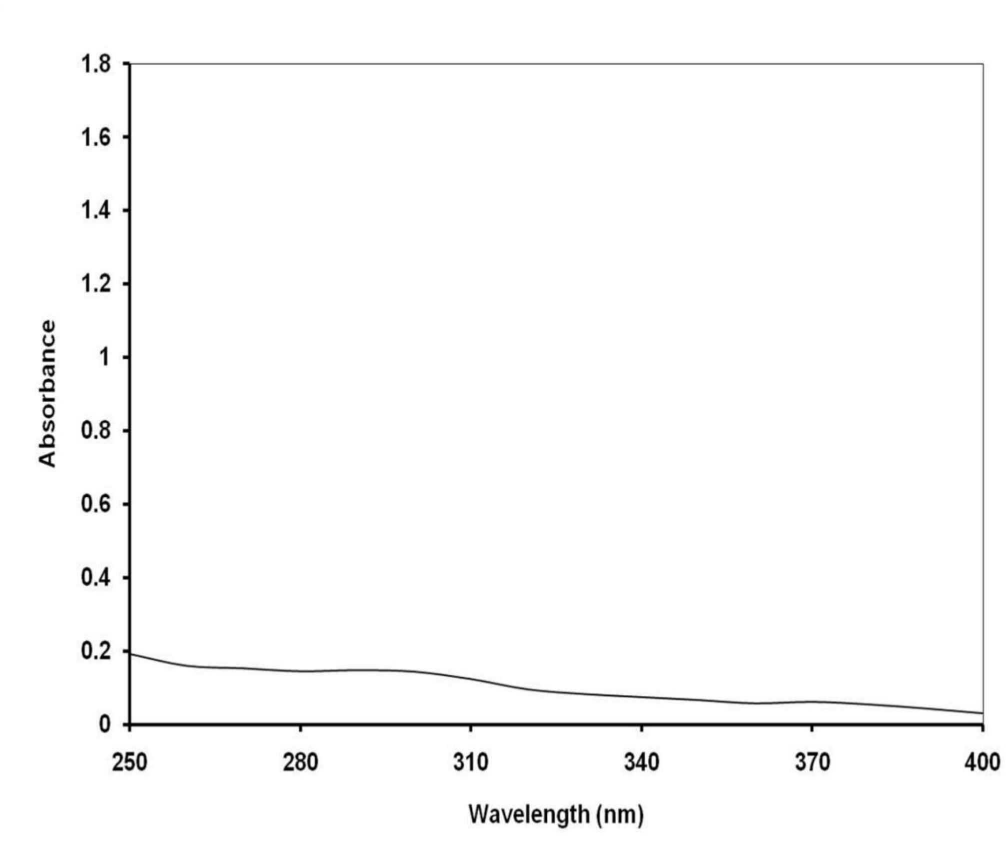

Figure S1. UV–vis absorption spectrum of Aloe vera leaf extract.

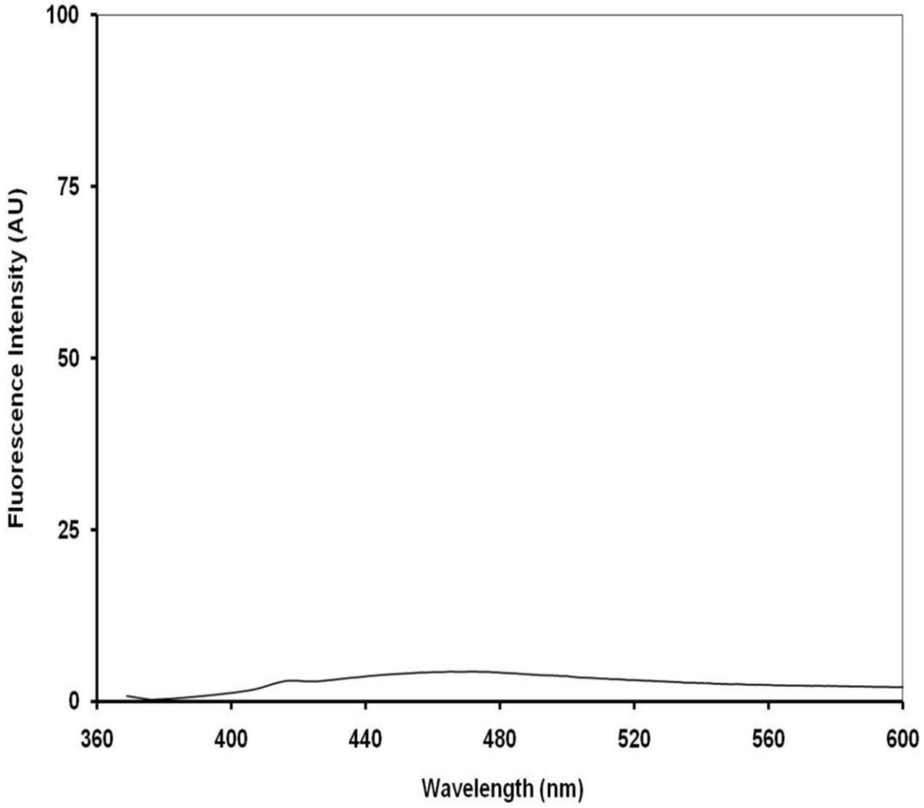

Figure S2. Fluorescence emission intensity profile of Aloe vera leaf extract. Fluorescence intensities were measured at excitation and emission wavelengths of 365 nm and 444 nm, respectively.

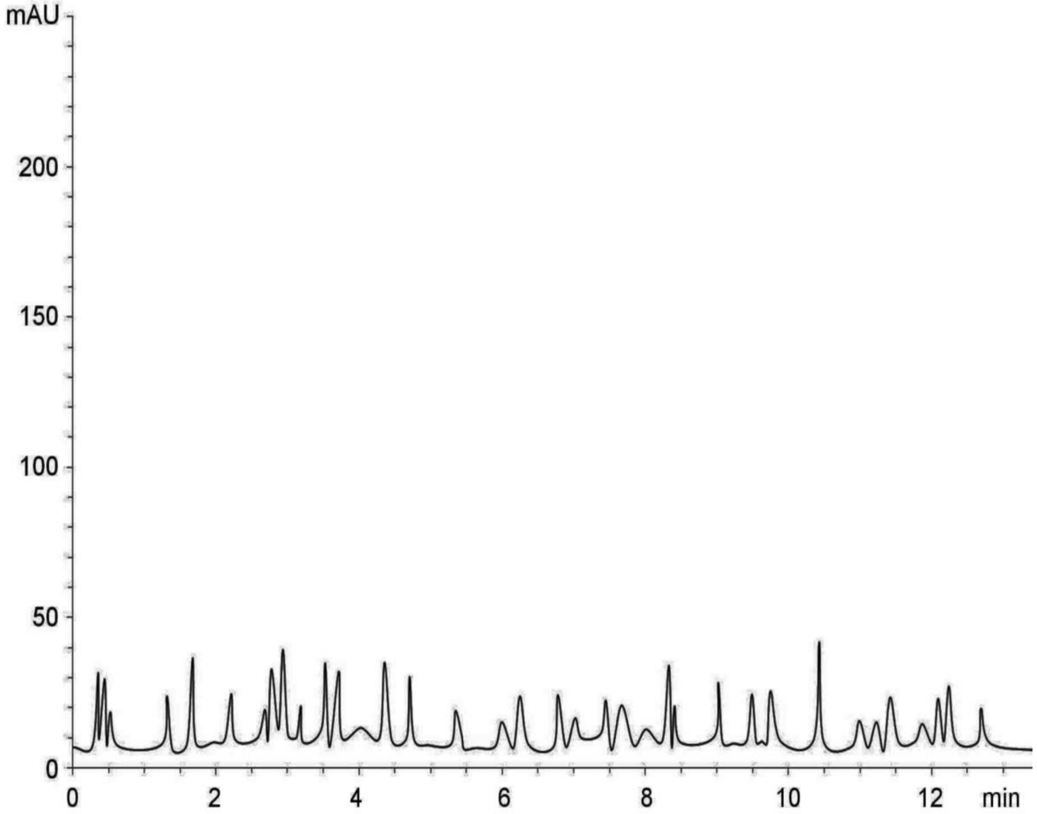

Figure S3. HPLC/UV elution profile of Aloe vera leaf extract.
